# A Treatise on the Nature, Causes, and Treatment of Erysipelas

**Published:** 1842-07-01

**Authors:** 


A Treatise on the Nature, Causes, and Treatment of Erysipelas. By *Thomas Nunneley*, Lecturer on Anatomy, Physiology, and Pathology, in the Leeds School of Medicine, &c. 8vo. pp. 307. London, John Churchill; Leeds, J. Y. Knight.

The principal object of Mr. Nunneley's book is to establish a class of erysipelatous inflammations, and to bring under this general head affections that have received very different names, and by many are considered totally different things. He remarks in his preface, "the word phlegmon has for long been generally employed as applicable to inflammations of various textures; and, in the same manner, the word erysipelas is now employed, as indicating a contrary condition or type, from phlegmon. If the term phlegmonous inflammation be allowed, he sees no reason why that of erysipelatous should not also be permitted. Indeed, it has already, by more than one writer, been so employed." He adds—"if decided opinions are expressed as to the intimate connection and relationship between the several affections mentioned, and that in nature they are the same as that spreading inflammation of the dermis, which is commonly denominated erysipelas, the author hopes the facts and arguments advanced in support of such opinions, will be thought, if not as convincing to others as to himself, at least sufficient to prevent the charge of advancing an unsupported hypothesis."

The affections that he places in the erysipelatous group are:—

I —Erythema, with its various species according to the classifications of Willan, Bateman, and Rayer; except the erythema intertrigo, fret, or erosion of the skin, which certainly has no relation to their other species of erythema. It is purely a local affection, produced solely by local irritants, wholly unconnected with constitutional symptoms, and not requiring constitutional remedies. It is also doubtful if erythema nodosum should be included: the local affection appears to be inflammation of the periosteum, the skin being only secondarily involved. How far the constitutional tendency and symptoms may approach to those attending erysipeloid affections is not quite certain.

II.—Erysipelas; in the forms commonly described under the term, whether of the head and face, trunk or extremities, idiopathic or sympathetic.

III.—Diffuse inflammation of the cellular membrane; as the term is used by Dr. Duncan, Jun. who first most distinctly called attention to this form of complaint.

IV.—Puerperal fever.

V.—Diffuse inflammation of the serous membranes; which is perhaps more frequently seen in the peritoneum; especially after wounds of it or in its neighbourhood, as after the operation for strangulated hernia or stone.

VI.—Diffuse inflammation of the mucous membranes; this form is more frequently seen about the fauces, as in some forms of angina pharyngea vel laryngea.

VII.—Very possibly some forms of ARACHNITIS belong to this class.

VIII.—DIFFUSE PHLEBITIS, and also this form of inflammation of the ABSORBENTS.

We would take the liberty of observing that whatever the truth, there cannot be said to be novelty in this view. Erysipelatous inflammation has become a household word in the profession, and not only tinctures our language but directly influences our practice. Nay, we believe that most well-informed persons include in the class of erysipelatous inflammations just the very affections that Mr. Nunneley has enumerated. While we cannot, then, acquiesce in the originality of this view, we are quite prepared to admit its truth, and we perfectly agree with Mr. Nunneley that any injuries of precisely the same nature, and in the same situation, which in one person shall induce a limited local action—phlegmon, either without much constitutional disturbance, or, if attended by general symptoms, they will be such as are indicative of power, in which depletants are not only borne, but are highly necessary; shall in another person, or even in the same at another time, induce a local action in which there is considerable tendency to spread far and wide—diffuse inflammation, or erysipelas, in which the constitutional symptoms are those of great action with little power, and where depletants are not only not indicated but are positively injurious. Farther, that these different states may arise without external injury, in which the local action may be exhibited upon the surface of the body, or be thrown upon an internal membrane, according as there may be some peculiar determinating cause in the part itself, or elsewhere.

Mr. Nunneley treats, in succession, of Erythema, Diffuse Inflammation of the Cellular Tissue, Puerperal Fever, Diffuse Inflammation of the Peritoneum and Pleura, Diffuse Inflammation of the Mucous Membrane, Diffuse Inflammation of the Arachnoid Membrane, Diffuse Inflammation of the Veins and Lymphatics.

Speaking of *Diffuse Cellular Inflammation*, Mr. Nunneley examines, of course, a very interesting and much mooted question; whether that form which follows dissection-wounds is the consequence of a specific poison or not. He observes:—

"From the fact of such serious consequences so much more frequently following wounds received in the examination of fresh bodies, than of those where putrefaction has already occurred, it might be supposed to depend upon some peculiar poison, generated during the course of the disease which has destroyed the person, did we not sometimes see the same effects follow clean flesh wounds, punctures, fractures, or bruises, and even occurring spontaneously.* While,

---

* "The following quotation from Dr. Carswell's Article on Mortification affords a strong support to what is advanced in the text,—'It is difficult to say how far wounds received in dissection, or in the inspection of dead bodies, and which are followed by diffuse erysipelatous and gangrenous inflammation, depend on the presence of a septic agent developed during the progress of disease, or after death; one thing is certain, that the frequency and severity of the disease which follows such wounds have, so far as we can perceive, no connection with the ordinary changes of the solids and fluids produced by putrefaction; for the results of our own experience are in accordance with the generally received opinion, however, this prevents us from at once adopting the idea of any such specific poison as will always induce the same disease as that by which the poison itself was originally produced, and which disease can only be so generated; there are a great number of facts to shew that the serous or semi-purulent matter thrown out during erysipelatous inflammation, and especially of the peritoneum, as shown in puerperal fever, has a much greater tendency to induce diffuse cellular inflammation, than any other kind of matter. But even this will not at all times, and under all circumstances, act in this manner. Much depends upon the state of health and constitution of the person inoculated; for, beyond all doubt, scores of anatomists are wounded in precisely the same manner, under precisely analogous circumstances, so far as the dead body is concerned, and altogether escape ill effects; and the same person may escape with impunity for a long series of years, and yet ultimately fall a sacrifice, as the case of Mr. Dease illustrates." 46.

It has always appeared to us, that they who argue questions of this kind are apt to lose sight of the consideration, *that* in order for a specific morbid poison to act upon a person exposed to its influence, it is necessary that the recipient should be in a certain state, or predisposed to it. We find this to be the case with syphilis, the exanthemata, typhus. The amount of predisposition requisite appears to vary greatly in different diseases. In some, as syphilis, it is slight, so that most persons exposed to its influence would suffer from it—in others, as typhus, it is considerable, so that few submitted to its influence become affected by it. If this be true, and we have no doubt of it, the circumstance of many anatomists pricking their fingers with impunity does not prove that they are not subjected to the operation of a poisonous matter; while the number of bad cases from dissection of bodies in a certain state, *does* prove that the matter applied exerts some operation independently of the condition of the individual that it is applied to. That the state of the individual is the most important element in the matter is evident, for it is when a season is drawing to its close, and students are suffering in health, that cases of dissection-wounds are usually observed

Passing on to *diffuse arachnitis*, we may cite Mr. Nunneley's notice of it. Certain it is, says he, that like the other serous membranes, the arachnoid is subject to the development of two forms of inflammation. In the one, there is intense excitement; a full, or more commonly, a hard incompressible pulse, frequent, but not excessively accelerated; and all the symptoms, both general and local, partake much of the nature of acute phlegmonous inflammation, as it occurs in other serous membranes, and require the same active depletion by bleeding and other antiphlogistic measures. In the other, the delirium is not attended with the same ungovernable excitement; there is a depression of the mental powers; the mind is rendered dull; in the former it is rather a perversion than a loss of mind; the pulse is rapid, neither full nor hard, or at least, if full not hard, but generally with a short hurried beat, which may easily be comthat it most frequently occurs after wounds received in the examination of recent bodies, and also in the bodies of those who have died of inflammatory effusions into the serous cavities.'—*Cyclopædia of Practical Medicine,* vol. iii. page 146."

pressed; and all the secretions are more deranged than in the former case,—especially the abdominal. In the former kind of inflammation the secretions are often suppressed, rather than excessively perverted, in the latter the perversion is the more prominent of the two; indeed, vomiting and diarrhœa are frequent accompaniments. The inflammation is much diffused, the effusion is more widely spread in the sub-arachnoid cellular tissue or pia mater, and it is of a sero-purulent nature. There is in the phlegmonous form, intense pain in the head, morbid sensibility to light, a contracted pupil, and much heat of the scalp; in the erysipeloid inflammation none of these are the usual prominent symptoms. The kind of persons and constitutions in which one or other of the forms appear, are marked by the same signs as point out predisposition to the development in other tissues of one or other form of complaint.

Alluding to the secondary deposits of pus which follow injuries of the head, Mr. Nunneley assures us that he is inclined to believe they will never, or rarely, be found to follow accidents to the head, unless preceded by diffused arachnitis; whether there may not also be phlebitis, and whether this latter be caused by the former, or the former by it, is not material, on account of the connexion between erysipelas and inflammation of the veins.

When treating of the causes of erysipelas, Mr. Nunneley refers to some facts illustrative of the influence of season and weather in its production. Out of thirty-eight cases which occurred in 1837, in the Dreadnought Hospital Ship, twenty-seven happened in the months of February, March, April,—September, October, and November, while in December and January only four cases occurred, the number of patients being greater during the Winter months, and in July and August only two. Perhaps of the two seasons, Spring and Autumn, more cases occur in the former, but the worst and most fatal in the latter. The effect of sudden change of the weather is forcibly instanced in the following fact:—In January, 1832, the weather for some time had been very open; on the 13th it suddenly changed, the wind blowing from the north-east, and very cold. On the following morning, three men who had wounds were seized with erysipelas, of which two died. One was in Luke's Ward, and two in Cornelius's Ward, Guy's Hospital, in neither of which had there been any cases of erysipelas for some time previously.

The constitution of the atmosphere which gives rise to the epidemic is not circumscribed to a very narrow limit, since it is rare for the disease to prevail exclusively in one hospital alone, though it may, from many causes, be more rife in one than in others. In Paris, in the Spring of last year (1840), erysipelas prevailed, not only in the various hospitals but also in the city. The same has been observed at Edinburgh, (when Dr. Duncan's cases of diffuse inflammation of the cellular membrane occurred), at Dublin, at Birmingham, and at Devonport, when Dr. Butter's cases of irritative fever prevailed.

Mr. Nunneley takes up the question whether erysipelas is or is not infectious. He argues, very justly, that it is. But perhaps the most interesting point is the relation of two cases of inoculation. One of them will sufficiently illustrate the fact. Mary Glisby was admitted an out-patient under his care as dresser at Guy's Hospital, in August, 1831.

She had an indolent bubo in the groin, which, after being seen two or three times, was dressed with red precipitate. In going home afterwards to Deptford, she got very wet: erysipelas appeared round the bubo, and spread over a large surface of the integuments. During this time her sister, who was in the habit of washing and dressing the sore, cut the thumb of the right hand: she took no precaution, but on the same day and almost immediately afterwards, washed the erysipelatous sore as usual. Within a few hours afterwards the cut inflamed and became very painful, and from it erysipelas spread over the whole arm.

*The Treatment of Erysipelas* is very elaborately considered by our author. After quoting authorities on the subject of *Venesection*, authorities of the most diametrically opposite description, he arrives at this conclusion:—"On the whole we must conclude that erysipelas is not one of those disorders for the cure of which venesection should form a prominent part of the treatment, and if not practised within a few days after the commencement of the attack, ought not, except under very extreme circumstances, to be practised at all; and I believe there are few cases in which the patient does not recover much sooner if general bleeding has not been practised than where it has. Bleeding is especially adapted for those disorders where it is of importance to produce an immediate effect upon the system, not only by the removal of a large quantity of the circulating fluids, but by the present and direct influence the sudden abstraction of the blood has upon the nervous system. Erysipelas is not a disorder of this kind, it cannot be removed in this way, and in the great proportion of cases, the fluids may be sufficiently, and much more naturally, removed in other ways." Our own experience has been town experience, and we must confess that the case in which bleeding has been called for or proved serviceable has not yet presented itself before us. And without dogmatically affirming one thing or the other, we cannot avoid entertaining a doubt whether venesection can be really required in erysipelas, be it in country or town. The very character of the inflammation does appear to us to be, *à priori*, opposed to very lowering remedies.

On the subject of *tonics* and stimulants we are much disposed to coincide with Mr. Nunneley, for we suspect that there has been as much extravagance on the part of the advocates of bark as on that of the patrons of bleeding. Mr. Nunneley observes:—"I can conceive of very few cases in which bark in any of its forms, or indeed any tonic or stimulant, can be proper at the onset of the disease, at least in adults; but it would seem, from the success attending the practice when the complaint occurs in infants, as well as from the symptoms sometimes manifested in these powerless beings, that even from the very commencement of the attack, bark or some of its preparations, especially quinine, ought to be largely administered. The symptoms which are usually present in the early stages of erysipelatous inflammation are such as are opposed to the administration of tonics, the irritable condition of the alimentary canal is such, that remedies of this class are decidedly contra-indicated; besides, there is not, with very few exceptions, at the commencement of erysipelas, such debility as to demand support, and there can be little doubt, as has been stated by Carmichael Smyth, that the administering of bark and wine at too early a stage of the disorder, has a great tendency to produce that very condition of putridity and gangrene which it is so much wished to prevent. So long as there is any considerable nausea and vomiting, it is impossible that these remedies can bestow any real power; bark and tonics only overload the stomach and add to its disorder, while wine and stimulants increase the headache and general irritation; but so soon as the tongue becomes clean and the stomach quiet, they certainly have a great effect in restoring the vigour of this organ.

"More commonly stimulants can be much earlier used and with more advantage than tonics, and especially stimulants of a diffusible kind, of which ammonia is certainly the best, as it appears to rouse and give energy to the nervous system, without acting upon the vascular, at least by increasing its excitement. At a later stage, when the tongue becomes dark or glazed, or the pulse weak and feeble, and the strength failing, then wine may be given with great advantage, in large quantities, or there may be substituted for it, with singularly good effect, the accustomed beverage of the patient, as porter or gin, the latter of which Sir A. Cooper speaks of in high terms. I think it will generally be found that these stimulants are of decided use, provided the tip and edges of the tongue are moist and not covered with sordes, however much the base and middle are loaded, but when the tongue is completely dry they either are not well borne, or are rejected,—a state in which turpentine may sometimes be most usefully employed instead." 222.

These *eclectic* sentiments will be found, we apprehend, not to stray wide of the truth. At the same time we believe that there are times and cases where diffusible stimulants are required even from the earliest stage —we allude to the erysipelas of the cachectic and sometimes of the aged. To wait for a certain period, or until the tongue puts on a certain state, will in such cases be sometimes found to wait too long, and to sacrifice what little chance there may otherwise have been.

We cannot shut the volume before us, without recommending it to our medical friends. It contains much information, and is marked by much good sense. The practitioner will find in it that which will stand him in stead in practice.

